# Application of large language models to the annotation of cell lines and mouse strains in genomics data

**DOI:** 10.1093/database/baag041

**Published:** 2026-07-22

**Authors:** Sanja Rogic, B Ogan Mancarci, Brianna Xu, Anna Xiao, Carlton Yan, Paul Pavlidis

**Affiliations:** Michael Smith Laboratories, University of British Columbia, Vancouver, BC, V6T 1Z4, Canada; Department of Psychiatry, University of British Columbia, Vancouver, BC, V6T 1Z3, Canada; Michael Smith Laboratories, University of British Columbia, Vancouver, BC, V6T 1Z4, Canada; Department of Psychiatry, University of British Columbia, Vancouver, BC, V6T 1Z3, Canada; Michael Smith Laboratories, University of British Columbia, Vancouver, BC, V6T 1Z4, Canada; Department of Psychiatry, University of British Columbia, Vancouver, BC, V6T 1Z3, Canada; Michael Smith Laboratories, University of British Columbia, Vancouver, BC, V6T 1Z4, Canada; Department of Psychiatry, University of British Columbia, Vancouver, BC, V6T 1Z3, Canada; Michael Smith Laboratories, University of British Columbia, Vancouver, BC, V6T 1Z4, Canada; Department of Psychiatry, University of British Columbia, Vancouver, BC, V6T 1Z3, Canada; Michael Smith Laboratories, University of British Columbia, Vancouver, BC, V6T 1Z4, Canada; Department of Psychiatry, University of British Columbia, Vancouver, BC, V6T 1Z3, Canada

## Abstract

Accurate, consistent and comprehensive metadata are essential for the reuse of functional genomics data deposited in repositories such as the Gene Expression Omnibus (GEO), however, achieving this often requires careful manual curation, which is time-consuming, costly and prone to errors. In this paper, we evaluate the performance of Large Language Models (LLMs), focusing on OpenAI’s GPT-4o, as an assistive tool for entity-to-ontology annotation of two commonly encountered descriptors in transcriptomic experiments, mouse strains and cell lines. Using over 9 000 manually curated experiments from the Gemma database and over 5 000 associated journal articles, we assess the model’s ability to identify relevant free-text entries and map them to appropriate ontology terms. Using zero-shot prompting and retrieval-augmented generation (RAG) to incorporate domain-specific ontology knowledge, GPT-4o correctly annotated 77% of mouse strain and 59% of cell line experiments, and uncovered manual curation errors in Gemma for over 200 experiments (2% of total). GPT-4o substantially outperformed non-LLM alternatives, and was statistically indistinguishable from the highest-performing 2026 frontier models. Model errors often arose from typographical mistakes or inconsistent naming in the GEO record or publication, and resembled those made by human curators. Along with annotations, our approach requests that the model output supporting context and verbatim quotes from the sources. These were typically accurate and enabled rapid curator verification. We further found that for the difficult cell line task, an ensemble of LLMs can boost precision at the cost of recall. These findings suggest that while LLMs are not ready to fully replace manual curators, they can effectively support them. A human-in-the-loop workflow, in which LLM’s annotations are provided to human curators for validation, should improve the efficiency and quality of large-scale biomedical metadata curation.

## Introduction

Biomedical databases and repositories are essential for gathering and organizing the vast amounts of generated data, ensuring that these resources are findable and accessible to the scientific community. One such repository is the NCBI Gene Expression Omnibus (GEO), which has accumulated hundreds of thousands of high-throughput functional genomic datasets spanning diverse technologies, organisms, tissues and experimental conditions [1]. These data are consistently used for secondary analysis, meta-analysis, method development and development of derivative databases. However, the reuse of the data critically depends on the availability of accurate and consistent metadata. While GEO has established submission formats and metadata standards, these are intentionally flexible and not strictly enforced to reduce the burden on data submitters. This can lead to missing, ambiguous or mistyped experiment information, discrepancies with the associated publication and broader inconsistencies across studies.

To make GEO datasets more findable, comparable and reusable, resources like Gemma [2] curate experiments and map free-text descriptions onto controlled vocabularies and ontologies, including terms for features such as organisms, strains, cell lines, drugs, genotypes, tissues, and diseases. Ontology-based annotation enables more effective database querying through ontology inference, facilitates data aggregation across studies, and simplifies downstream computational analyses. However, manual curation is resource-intensive and does not eliminate the risk of errors.

In this study, we evaluate the ability of GPT-4o to annotate two types of entities that are often encountered in metadata of transcriptomic experiments: mouse strains and cell lines. This task is difficult because it includes both a concept identification task (‘Is this the actual strain used in the genomics experiment vs. just a passing mention?’) and a normalization task (‘What is the ontology identifier for the cell line?’). Large language models (LLMs) offer a potential way to expedite and facilitate the curation process due to their ability to interpret natural-language inputs and find key concepts within complex writing.

Unlike approaches that rely primarily on string similarity and predefined lexicons, LLMs use contextual cues to disambiguate entities. Recent models such as GPT-4o can process long heterogeneous inputs and follow complex instructions, making them good candidates for data curation tasks. Previous work showed promising results for a range of data annotation and information extraction tasks [3–10], but these studies were typically limited in scope, either relying on sentence-level inputs or evaluating performance on only a few dozen scientific articles.

Here, using over 9 000 manually curated experiments from the Gemma database as a reference, as well as over 5 000 associated journal articles, we assess GPT-4o’s performance in identifying the relevant free-text entries from GEO metadata and linked publications and mapping them to domain-specific ontology terms. We employ a zero-shot prompting strategy and use a retrieval-augmented generation (RAG) approach [11] to ground the model with pre-compiled lists of ontology terms. The idea behind RAG is, before querying the LLM, to first identify information that would help the query (retrieval) and include it (augment) in the prompt. Our findings provide a systematic assessment of GPT-4o as an assistive tool for ontology-based curation of transcriptomic experiments and highlight both the promise and current limitations of LLMs for large-scale biomedical metadata annotation.

## Methods

### Compiling domain-specific knowledge

To provide GPT-4o with relevant external domain-specific knowledge, we compiled separate lists of ontology terms for each task. As our project was aimed at application in the Gemma system [2], we used ontologies already in use, including our small in-house ontology, TGEMO (https://github.com/PavlidisLab/TGEMO). For the mouse strain identification task, we used the Experimental Factor Ontology (EFO, Malone *et al*. [12]) and the Gemma Ontology (TGEMO) to extract all child terms (classes; i.e. mouse strains) of the term ‘Mus musculus’ (http://purl.obolibrary.org/obo/NCBITaxon_10090). This resulted in a list of 156 commonly used mouse strains. For the cell line identification task, we combined all terms (classes) from the Cell Line Ontology (CLO, Sarntivijai *et al*. [13]) with the child terms of ‘cell’ from EFO (http://purl.obolibrary.org/obo/CL_0000000). After removing duplicates, the final list included 46 032 unique cell lines. EFO and CLO are two widely used ontologies for genomics data annotation.

### Prompt design and annotation workflows

For all our primary analyses, we used OpenAI’s GPT-4o model (gpt-4o-2024–11-20) via its API and the OpenAI Python library (version 1.54.1). To improve reproducibility, the temperature parameter was set to 0 and the seed was set to 1.

For each transcriptomic experiment in the test dataset, we first generated a core prompt, consisting of a plain-text description of the task, followed by the experiment’s metadata sourced from GEO in JSON format. The central instruction in the prompt was (one version for each task): *‘Your task is to identify {mouse strains|cell lines} used in a differential expression experiment’*, stressing that *‘Descriptions of some experiments may include {strains|cell lines} that are not used for the differential expression analysis. If a {strain|cell line} is clearly not used for the differential expression analysis, do not return it*’. The metadata included the experiment title, summary and overall design, as well as characteristics and protocol descriptions for each individual sample. If the experiment has an associated publication that is publicly accessible through PubMed Central, we appended its title, abstract and Methods section. The full prompts are provided in our GitHub repository.

**Figure 1:**
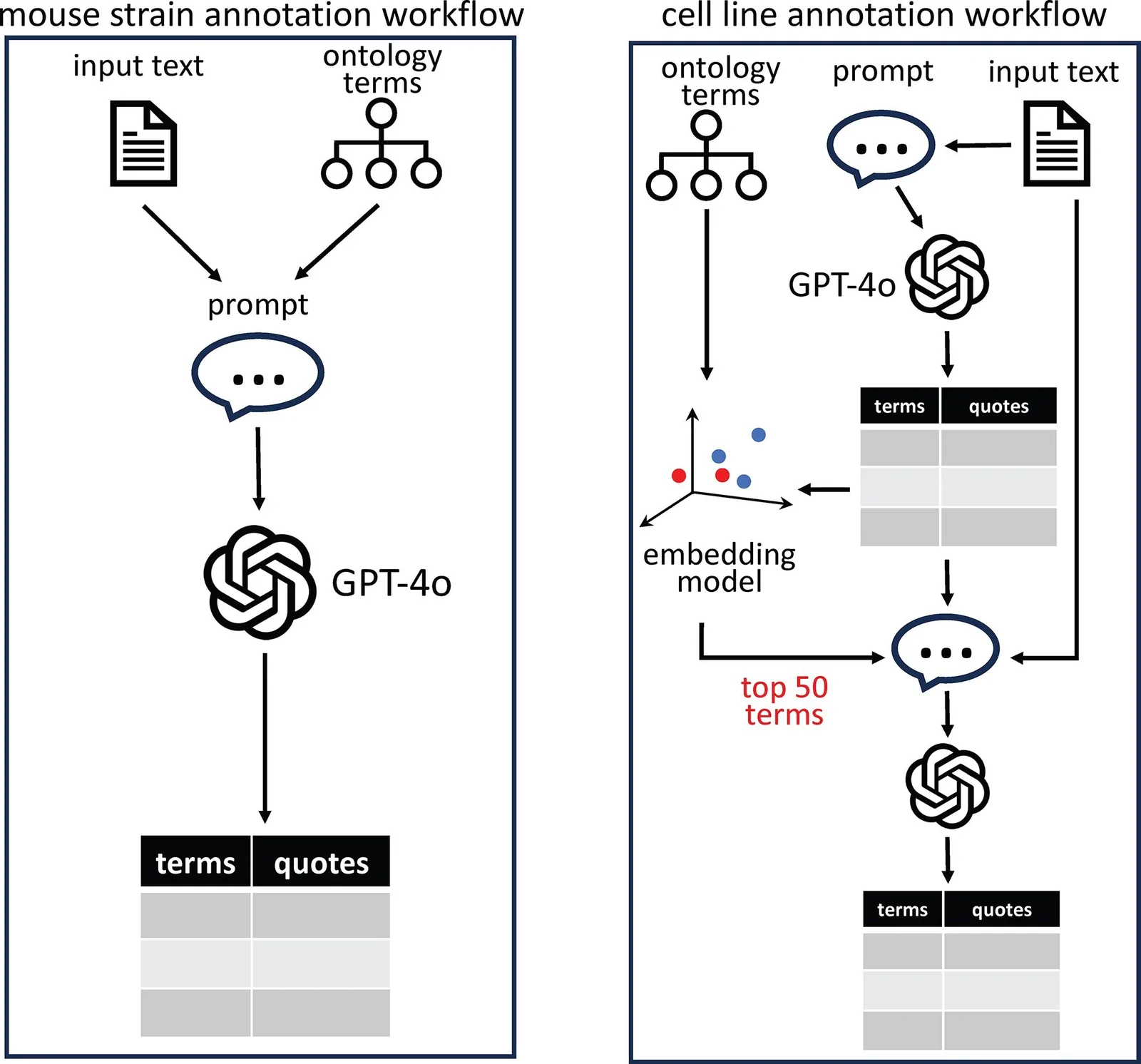
Curation workflows for the two types of annotation tasks. Each workflow begins with a prompt and input text consisting of the experiment’s metadata from GEO (title, summary, overall design, sample-level characteristics and protocol descriptions) and, when available, associated publication (title, abstract and Methods sections). (a) The input for the mouse strain task also includes a list of ontology terms (labels, URIs and descriptions). GPT-4o is prompted to return the labels and URIs of all mouse strains detected in the input, along with verbatim quotes supporting each decision. (b) Annotation of cell lines is a two-step process. First, GPT-4o is prompted to return cell lines identified in the input as free-text entries, together with supporting quotes. Next, these responses are embedded and compared against a pre-generated vector database of cell line ontology terms, and the top 50 most similar terms are returned to GPT-4o along with the original input text and first-stage outputs. Finally, GPT-4o is instructed to select the corresponding ontology term.

For the mouse strain identification task, the prompt additionally included URIs and descriptions of compiled ontology terms, provided as input labels in JSON format. The prompt instructed the LLM to produce output conforming to a predefined JSON schema to enable programmatic processing and to return the labels and URIs of all identified mouse strains, together with verbatim quotes from the source text supporting each annotation decision (Fig. 1a).

The cell line annotation task was performed in two stages using separate rounds of LLM prompting (Fig. 1b). First, GPT-4o was asked (via the core prompt described above) to identify cell lines mentioned in the input text and to return them as free-text entries, together with verbatim quotes supporting each identification. Directly mapping these mentions to ontology terms within a single prompt was not feasible because GPT-4o’s context window (128k tokens) could not accommodate the complete set of 46 032 cell line ontology terms.

To address this limitation, all ontology terms and their descriptors (names, synonyms and definitions) were embedded using the ‘text-embedding-3-large’ model to create a vector database. The free-text cell line mentions generated in the first stage were then embedded and compared against this database. For each mention, the 50 most similar ontology terms were retrieved based on cosine similarity.

In the second stage, GPT-4o was provided with the original input text, the first-stage outputs, and the 50 candidate ontology terms retrieved for each cell line mention, together with their cosine-similarity rankings. GPT-4o was then instructed to replace the free-text mentions with the most appropriate ontology labels and URIs.

### Non-LLM methods

We evaluated several other approaches. The output was evaluated using the same criteria as for the GPT-4o’s evaluation, but any additional mismatches with Gemma’s annotation generated by these methods were not manually reviewed. Except where noted, these analyses used a single random sample of 500 experiments rather than the full corpus.

#### Regular expressions

For the strain task only, we used a string-matching method based on regular expressions to search for ontology terms or their synonyms from the task-specific ontology lists within the same input text provided to GPT-4o. To minimize random or spurious matches, we excluded all terms shorter than three characters. We also removed terms that commonly appear as ordinary English words in the text, such as NOR (used for the NOR mouse strain) or CAST (a synonym for the CAST/EiJ mouse strain). An annotation was assigned to an experiment when a case-insensitive match was detected.

#### Text2term

We ran the term-frequency/inverse document frequency (TFIDF) mapper text2term using the same 156-term mouse strain dictionary. The dictionary was synthesized as a minimal OWL document so that text2term’s back-end can parse it. Candidate strings were extracted from the same fields as the regex baseline searches (characteristics, study summary, paper abstract/methods). Predictions were retained at TFIDF similarity ≥ 0.4 with at most one mapping per candidate string. Note that this value was overfit because we were surprised at text2term’s poor performance and tried different thresholds, so the performance reported for text2term is slightly optimistic.

#### BM25

BM25 [14] is a standard baseline. The index was built once over each ontology term’s value + synonyms (description excluded; BM25 length normalization amplifies long descriptions unfairly); tokenization is case-folded alphanumeric runs. Per-experiment, the full input document (*overall_design + summary + per-sample characteristics + paper text when available*) was tokenized the same way and scored against all 156 terms; we took the top selected term.

#### SapBERT

We ran SapBERT (Liu *et al*. [21]; checkpoint cambridgeltl/SapBERT-from-PubMedBERT-fulltext) as a non-LLM neural reference on both tasks. Each ontology term (name and every synonym) was mean-pooled from the model’s last hidden state, L2-normalized, and cached. For each experiment, we assembled a set of candidate input strings as follows: each sample-level characteristic value as one candidate; the study summary, overall design and (when available) the paper title as single candidates; and the per-sample protocol, paper abstract and paper methods split at sentence boundaries, and each sentence capped at 300 characters. The candidate set was capped at 80 strings per experiment. Every candidate was embedded in a single batched forward pass, compared by cosine similarity against every cached dictionary embedding, and assigned to its top-1 nearest dictionary term if that score exceeded 0.85. The prediction was the union of accepted terms across all of its candidates.

### Additional evaluations

These were also based on the same samples of 500 studies for each task as used for the non-LLM methods.

#### Alternative LLMs

The prompts were the same as those used for the GPT-4o analysis. All Claude inference used the official Anthropic Python SDK (v0.102.0). Max_tokens was raised to 2 048 (strain task) from the 1 024 used for GPT-4o to accommodate Claude’s marginally more verbose JSON formatting. Temperature was set to 0 for Sonnet 4.6 and Haiku 4.5. Opus 4.7 does not accept a temperature parameter. Structured output was enforced with Anthropic’s tool-use mechanism. We additionally ran Llama 3.3 70B Instruct as an open-weights baseline on the same 500-experiment mouse strain sample. Inference used meta-llama/Llama-3.3–70B-Instruct-Turbo served through Together AI’s OpenAI-compatible HTTP API (https://api.together.xyz/v1), with the OpenAI Python SDK pointed at that base URL. Inference settings were otherwise identical to the Claude runs (max_tokens = 2048, temperature = 0, system prompt and 156-term strain list verbatim).

#### Hybrid dense + sparse retrieval (cell line task)

As an alternative to RAG from dense embeddings, we evaluated a hybrid retriever combining the existing dense (OpenAI `text-embedding-3-large`) and a BM25 sparse channel via reciprocal rank fusion. Each channel returns its top-200 per query; the fused score for an id is the sum of `1/(60 + rank_in_that_retriever)` across the two channels (a candidate that does not make either retriever’s top-200 contributes 0). The top-50 by fused score is then handed to the Stage-2 LLM. The Stage-1 extractions, the Stage-2 prompt, model parameters, and the evaluation rule were kept the same.

#### Sensitivity analysis for embedding-based RAG (cell line task)

To evaluate the effect of the amount of retrieval-augmented context provided to the LLM, we repeated the second stage of the cell line annotation task using K ∈ {10, 25, 50, 100, 200} top retrieval hits on a random sample of 100 datasets from the 500. The first-pass extractions and OpenAI embeddings were reused from the main Sonnet 4.6 run; only the candidate window and the second-pass prompt were reissued.

#### Sensitivity analysis of the effect of publication availability

To quantify the contribution of the linked publication to annotation accuracy, we ran an analysis of 291 of the 500 strain-task datasets for which a PubMed-Central paper was retrievable. The same GPT-4o + specificity-rule setup was issued twice per experiment: once with the default input (GEO metadata + the paper’s title, abstract, and methods section concatenated as a single paper’s field in the user message), and once with the paper’s field omitted.

#### Accuracy of supporting quotes

We tested whether the supporting evidence reported by the LLMs was accurate by testing whether each emitted quote was a substring of the input, for the same sample of 500 studies. ‘Strict’ verification asks whether the quote is a case-folded, whitespace-collapsed substring of the input. ‘Normalized’ verification additionally strips every non-alphanumeric character before checking, so cosmetic differences (en-dash vs hyphen, added terminal periods, multi-line joining) collapse to a single class. The gap between the two rates per model is a measurement of how much of the ‘unverified’ quotes are paraphrases or hallucinations, versus pure character-set normalization.

#### MetaMuse (cell line task)

We adapted the software of Mittal *et al*. [15] to work with OpenAI’s API and used GPT-4o (instead of Gemini as used by Mittal et al.), and ran it using the same 500 datasets and text inputs as for the other added evaluations of the cell line task. MetaMuse processes individual GSMs (samples), not GSEs (datasets). To avoid excessive LLM costs, we capped the run at 3 random GSMs per GSE (we note that this is a much larger dataset than used by the MetaMuse authors, who evaluated on 100 samples in their main analysis). This random sample selection was slightly suboptimal, because it’s possible for two GSMs to share the same cell line metadata, making them redundant, but we estimated the impact of this on our results to be minimal, as the experiment-level metadata is provided each time regardless. Parameters for MetaMuse were: conditional_mode = eval, max_iterations = 2, batch_size = 10, max_workers = 3. The final call is the set-union over the cell line predictions made for the 3 GSMs. Evaluation followed the same approach as the rest of the work, including the cross-walk step.

### Experiment curation in Gemma

The Gemma manual curation procedure is described in Lim *et al*. [2]. We provide a brief overview here for context. The process in Gemma begins by automatically parsing and uploading the experiment’s metadata from GEO SOFT files. All experiments are then subjected to careful manual curation performed by trained undergraduate research assistants who follow an established set of guidelines designed to ensure consistency across datasets. The curation process captures both the experimental design and the scientific topics of each study by mapping free-text metadata and publication descriptions to controlled vocabulary terms.

Gemma uses ontology-based annotation to assign category–value pairs to relevant biological entities in the metadata. Examples of categories include tissue or organ, disease, treatment, sex, mouse strain and cell line. Gemma supports several established biomedical ontologies for annotating values within these categories, of which EFO for mouse strains and CLO for cell lines are most relevant to this paper. Built-in curation tools assist curators in selecting appropriate ontology terms by suggesting previously used terms and encouraging consistent usage. Annotations may be reviewed by a second curator and periodically audited to ensure quality and consistency.

### Evaluation of automatic annotations

We first performed an automated comparison between the annotations produced by GPT-4o or other approaches and the existing Gemma annotations. Experiments for which the predicted ontology terms exactly matched the corresponding Gemma terms were classified as correctly annotated. For all remaining experiments, curators (N = 1 per prediction; authors BX, YX and CY) manually reviewed discrepancies to determine the source of the mismatch and, when necessary, corrected Gemma annotations before finalizing their assessment. A case was counted as ‘correctly captured’ when the agent’s free-text extraction identified the same strain entity intended by the curator, including all components of compound strains, even when the resolved URI differed from the correct one or when free text was emitted in place of a URI.

To quantify predicted annotation accuracy, for each experiment, we computed the following metrics:

- True Positives (TP)—number of correctly predicted ontology terms
- False Positives (FP)—number of incorrectly predicted ontology terms, i.e. not present in Gemma’s annotation
- False Negatives (FN)—number of ontology terms present in Gemma’s annotation that were missed by the evaluated approach

Based on these metrics, we computed the following performance measures for each experiment:


\begin{eqnarray*}
{\mathrm{Recall\ }} = {\mathrm{\ }}\frac{{TP}}{{TP + FN}}
\end{eqnarray*}

\begin{eqnarray*}
{\mathrm{Precision\ }} = {\mathrm{\ }}\frac{{TP}}{{TP + FP}}
\end{eqnarray*}

\begin{eqnarray*}
{\mathrm{F}}1{\mathrm{\ Score\ }} = {\mathrm{\ }}\frac{{2\textit{xRecallxPrecision}}}{{\textit{Recall} + \textit{Precision}}}
\end{eqnarray*}

To ensure meaningful estimates, experiments for which the evaluated approach produced no annotations (TP + FP = 0) were omitted from the computation of average precision. In some figures, we report accuracy, the proportion of experiments that were correctly annotated.

#### Statistical comparisons of methods

We compared methods run on the same corpus with the McNemar test with Edwards continuity correction [16] on the binary correctness outcome. We report raw % agreement on the correct/wrong outcome and Cohen’s kappa [17] which corrects for chance agreement. We report 95% confidence intervals computed using the method of Wilson [18].

## Results

Our initial experiments used GPT-4o (released in May 2024), which was the most advanced OpenAI model available during the initial study period. Using the model’s API, we annotated two types of information commonly found in GEO’s metadata for transcriptomic experiments: mouse strains and cell lines. This combines the tasks of identifying which strain/cell line was actually used in the transcriptomic experiment, and then normalizing that concept to an ontology term. We evaluated performance using experiments from Gemma, which have been manually annotated with ontology terms from these categories. Gemma’s data curation and annotation are performed by trained undergraduate research assistants, and various internal checks and reviews are used to ensure high accuracy; however, errors can occur.

**Figure 2:**
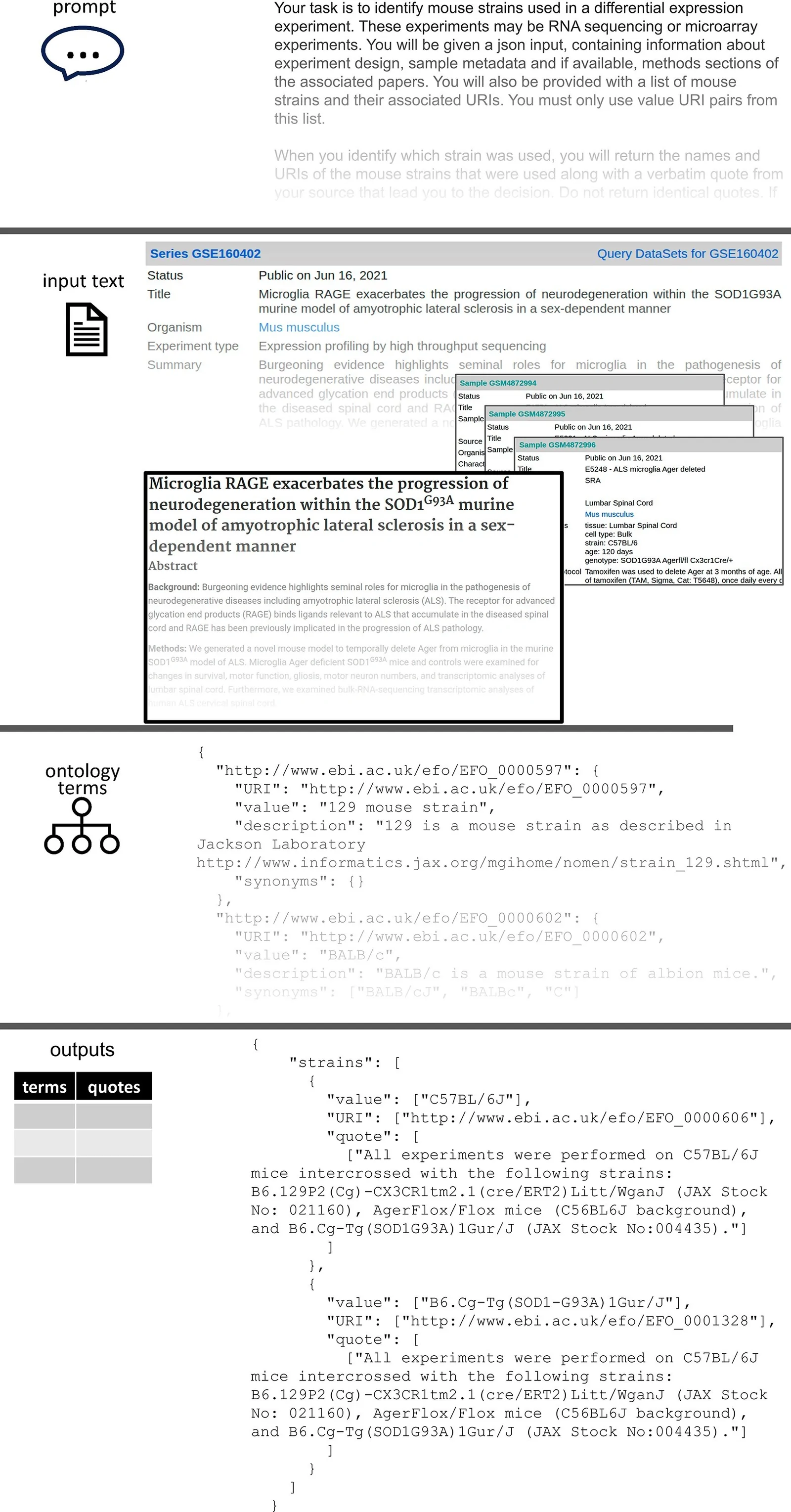
Example of the mouse strain annotation workflow. An example given for GEO experiment GSE160402, including the prompt, input text, ontology term list (in JSON format), and the resulting output (in JSON format).

For each experiment, we constructed a prompt that included the GEO metadata and, when available, the title, abstract and Methods section of the associated publication. No examples of correct annotations were provided, consistent with the zero-shot prompting approach. We applied a Retrieval-Augmented Generation (RAG) approach to ground the LLM with domain-specific knowledge, using pre-compiled lists of ontology terms relevant to each task. To facilitate evaluation, GPT-4o was instructed to produce programmatically consumable output along with supporting evidence for each annotation (Fig. 2).

### Annotation of mouse strains

For the mouse strain annotation task, we selected a set of previously curated transcriptomic experiments from Gemma, using the existing Gemma annotations as the baseline. We included experiments annotated with at least one ontology term from the task-specific lists described above, yielding 6 013 experiments in total, 3 362 of which had associated publications (Table 1). The automated comparison between GPT-4o and Gemma annotations yielded 4 214 exact matches. Manual inspection of the remaining 1 799 experiments identified an additional 422 cases in which GPT-4o correctly captured all relevant mouse strains, bringing the total number of correctly annotated studies to 4 636 (77%). Manual review also revealed 230 studies in which Gemma’s annotations were inaccurate; these were corrected prior to completing the evaluation. Among the 1 377 studies where GPT-4o’s output still disagreed with the corrected Gemma annotations, most discrepancies were due to GPT-4o missing some strains (1 286), while in 893 cases it also added incorrect annotations. Overall, average (± standard deviation) recall was 0.82 ± 0.36 and average precision was 0.82 ± 0.36. The mean F1 score was 0.82 ± 0.36.

**Table 1:** Summary of task-specific evaluation datasets and corresponding performance results.

	Experiments analyzed (with publication)	Manually reviewed experiments	Accurately annotated experiments	Experiments without false negatives	Experiments without false positives	Mean recall	Mean precision	Corrections of existing annotations
Mouse strain annotations	6 013 (3 362)	1 799	4 636 (77%)	4 727 (79%)	5 120 (85%)	0.82	0.82	230
Cell line annotations	3 377 (1 868)	1 063	1 990 (59%)	2 083 (62%)	2 215 (65%)	0.72	0.72	17

### Annotation of cell lines

GPT-4o’s performance on the cell line annotation task was evaluated using a similar procedure. Among 3 377 evaluated studies (1 868 with associated publications), GPT-4o produced perfect annotations for 1 990 of them (59%) (Table 1). Of the remaining 1 387 studies, 1 294 contained spurious annotations and 1 162 had missed annotations. The per-study accuracy metrics were: mean recall = 0.72 ± 0.43, mean precision = 0.72 ± 0.43, and mean F1 score = 0.72 ± 0.43. The lower accuracy compared to the strain annotation task likely reflects the substantially larger cell line ontology dictionary (46 032 terms) and high lexical complexity of the cell line ontology labels.

### Detection of human curation errors

GPT-4o’s output helped correct existing Gemma annotations in 230 studies for mouse strains and 17 for cell lines. For mouse strains, most curation errors stemmed from selecting the incorrect version among closely related strains, often due to inconsistent reporting within GEO metadata and/or the associated publication. For example, the GEO sample-level metadata for experiment GSE135602 specifies the mouse strain as FVB. However, the Methods section of the corresponding paper [19] identifies the strain more precisely as FVB/N, which our curators missed. Similar inconsistencies can occur within different sections of GEO metadata: for instance, the record of GSE127190 lists C57BL/6J in its overall design, but C57BL/6 in its sample metadata. Overall, at least 37% of the 247 (230 + 17) cases had such inconsistencies. Although improvements in curator training and curation procedures could potentially mitigate such issues, GPT-4o’s ability to quickly analyze the entirety of provided information proved especially effective in identifying and correcting these inconsistencies.

### Characterizing GPT-4o annotation errors

A closer examination of GPT-4o’s errors revealed that they were often similar in nature to those made by human curators. Many arose from typographical errors or alternative spellings in GEO metadata or in the associated publication. For instance, GPT-4o failed to return the mouse strain C57BL/6 for dataset GSE55188 because it was incorrectly referenced by the submitter as ‘C57/Bl6’. Similarly, for dataset GSE56690, the model returned C57BL instead of C57BL/6, most likely due to the misplaced slash in the strain name in the GEO record (C57/BL6). In general, we observed that GPT-4o was more likely to make mistakes in experiments where string matching failed to detect the correct strains than when it succeeded (25.6% vs 20.2% error rate; one-sided Fisher’s exact p = 6.5 × 10⁻⁶), suggesting that these cases present challenges even for context-aware models.

We also observed some hallucinatory errors, where the model produced terms not present in the input text; for example, for dataset GSE59018, GPT-4o reported FVB/NJ despite the only strain mentioned in the input text being FVB. Notably, however, in such cases the supporting quotes returned by the model were consistently faithful to the source text and always contained the correct term. This allows a human curator to quickly identify the error and make the necessary correction.

Using the two-step querying approach for cell lines allowed us to work with a much larger list of ontology terms, but it also introduced additional sources of error in the annotation process. We observed failures in both steps: either the model failed to identify valid cell line mentions in the input text or the correct ontology term was not retrieved among the top 50 candidate terms from the embedding-based similarity search. Errors in the first stage typically arose because entity identification was performed without ontology-constrained candidate terms. For example, for dataset GSE1977 GPT-4o mistakenly identified ‘NoCa’ as a cell line name, which the authors used as a designation for patients with no cancer. Dataset GSE41790 is an example of failure in the second stage, where the correct cell line, ‘BJ cells’, was identified initially, but the corresponding ontology term was ranked 87th based on the similarity search and therefore not provided to GPT-4o for mapping; instead, ‘BJ-derived cell line’ was selected as the final output. However, even when the correct ontology term was present among the top 50 candidates, GPT-4o did not always select it. For dataset GSE44157, the ‘murine mesenchymal stem cells’ cell line was correctly identified in the input text and the appropriate ontology term (‘mesenchymal stem cell line’) was ranked 13th based on the embedding space similarity search, yet GPT-4o selected the specific mesenchymal cell line ‘RCB1991’ despite the lack of supporting evidence.

### LLM-based annotation outperforms alternatives

**Figure 3:**
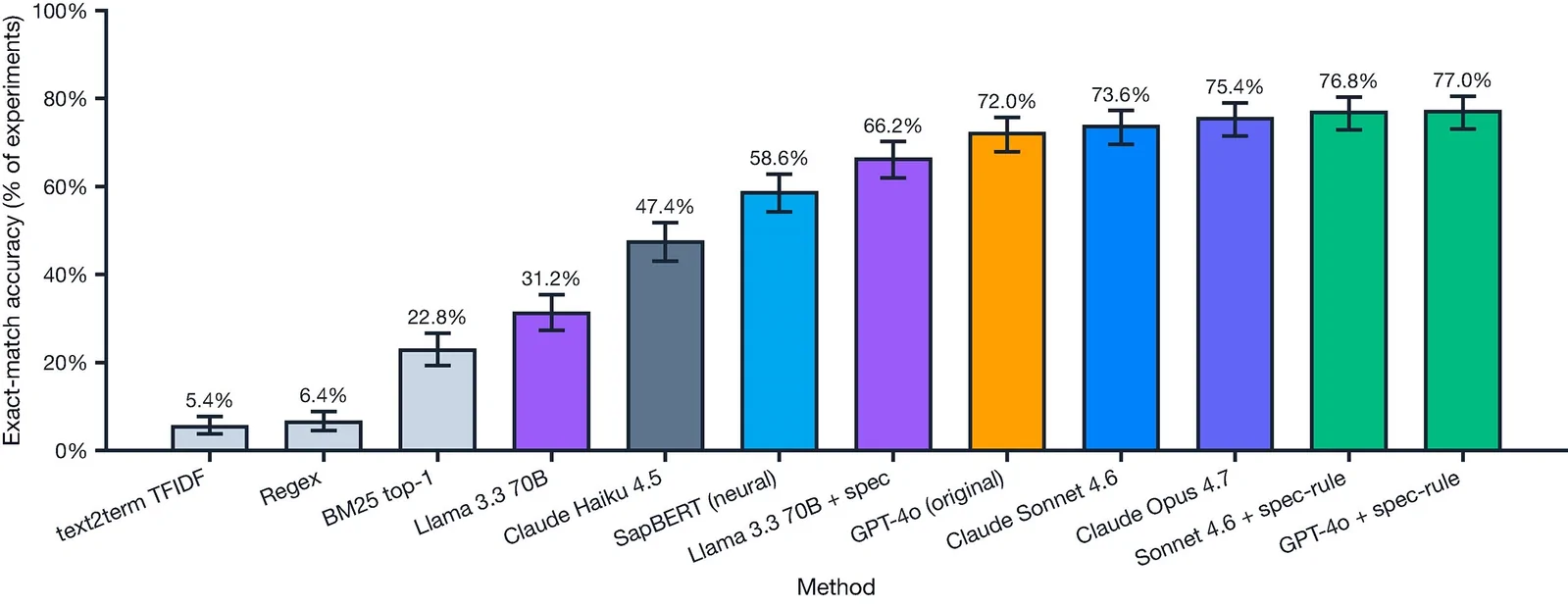
Strain annotation accuracy across methods. Twelve methods (regex, TFIDF, sparse-lexical, neural and frontier LLMs) were evaluated on the same 500-experiment sample. Error bars are Wilson 95% confidence intervals. Open-weights bars in violet; specificity-rule prompt variants in green.

**Figure 4:**
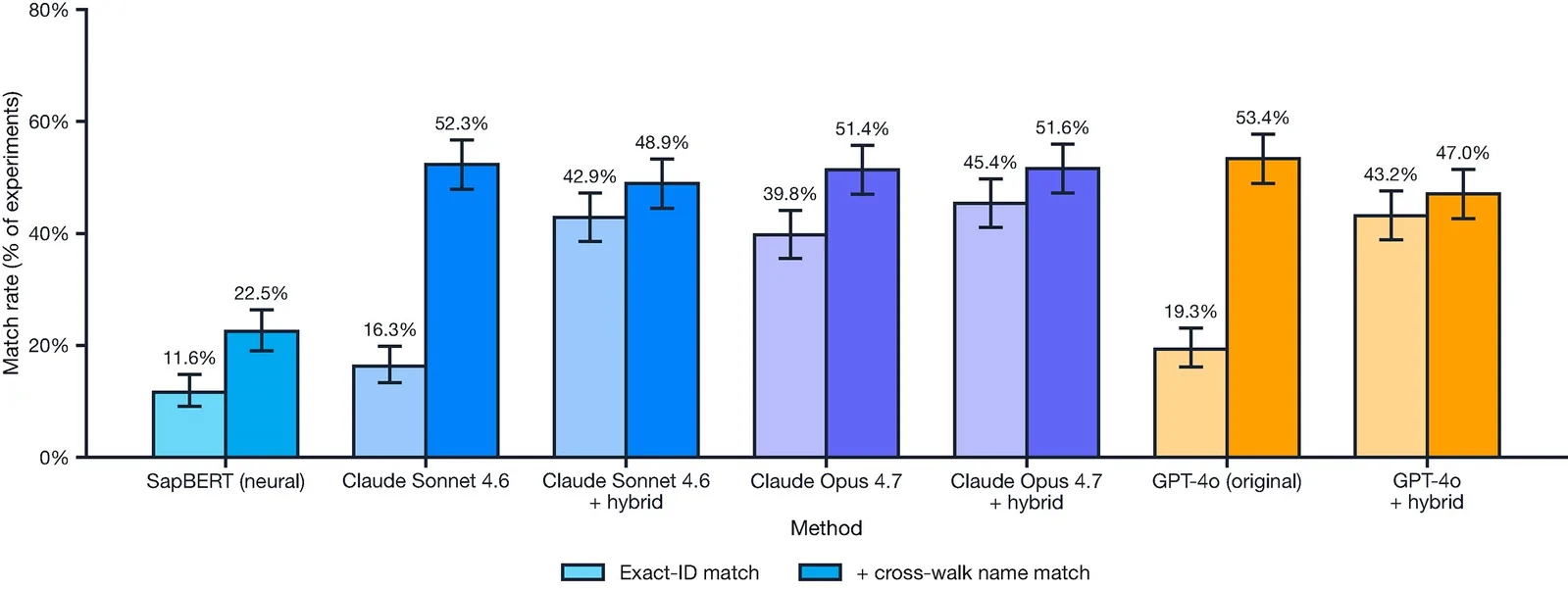
Cell line annotation accuracy across methods. Same 500-experiment cell line sample (n = 491–498 with usable rows). Two values per method: exact ontology-ID match, and matches after resolving EFO terms to their CLO equivalents via the cross-walk. ‘+ hybrid’: dense + BM25 + reciprocal rank fusion at retrieval; same Stage-2 model and prompt. Error bars are Wilson 95% confidence intervals.

We next compared our baseline GPT-4o approach to a range of alternatives (see Methods), including regular expressions, text2term [20], BM25 [14], SapBERT [21], and four additional LLMs: an open weights model (Llama 3.3 70B) and current models from Anthropic, Inc. (Haiku 4.5, Sonnet 4.6 and Opus 4.7). All of these were applied to the mouse strain task; for the cell line task, we tested two Anthropic models and SapBERT. For strains, we also tested a hybrid method that combines BM25 and the dense OpenAI embeddings, as there was a report that this combination performs well [15,22]. For each task, the evaluations were conducted on a random sample of 500 experiments from our corpus. Results are shown in Fig. 3 (mouse strains) and Fig. 4 (cell lines); precision and recall are shown in Supplementary Fig. S1 (strain micro-PR) and Supplementary Fig. S3 (cell line PR). Overlap in per-experiment errors across the four frontier strain models is shown in Supplementary Fig. S2. The main finding is that LLM methods outperform other approaches, with SapBERT only being competitive with Haiku. Regular expressions, text2term and BM25 were essentially unable to perform the task. There was no statistical difference between GPT-4o, Sonnet and Opus. The hybrid dense-sparse retrieval did improve performance on exact matches for strains, but this gap was closed by the trivial deterministic mapping of terms in EFO to the CLO equivalents.

We investigated sources of variability and avenues to improve LLM performance. One source of variability can be from the models themselves. In particular, the Opus 4.7 model does not accept a temperature parameter, which means its results are not guaranteed to be reproducible. To quantify inference-time stochasticity, we reran Opus 4.7 a second time on a 20-experiment strain subset, revealing that exact match accuracy for the strain task can swing ∼5% between runs. We interpret these findings as implying that differences between models of a few percentage points are unlikely to be meaningful (and indeed were not statistically significant here).

We next considered if the additional models might have uncovered more cases where accuracy was impacted by gaps or errors in Gemma. A curator reviewed 156 of the new cell line predictions across 58 datasets; 45% (71/156) were judged correct, 78 were judged wrong, and 7 were uncertain. Because this set of 156 is not representative of the full set of predictions, we present this as an extension of our findings in Supplementary Fig. S4. This does not change the overall pattern of the data, but again hints that our raw measures of performance may be conservative due to the imperfect gold standard.

We conducted additional sensitivity analyses. We first hypothesized that the presence of a publication associated with an experiment might have an impact. We used the 291 experiments in the 500-experiment sample for which a PubMed-Central paper was retrievable to run GPT-4o with or without text from the publication (see Methods). Performance on the 291 rose from 72.2% exact matches to 75.9% when paper information was used, though this was not statistically significant (p = 0.054). There were cases where adding the paper caused errors (8/291), apparently because other mouse strains were mentioned in the paper that distracted the model. For the cell line task, we also tested whether varying the number of candidates retrieved from the embedding had any effect. The effects were small until the number of hits was reduced to less than 20 (Supplementary Fig. S5).

As mentioned above, we noticed a common failure mode was the model picking a more specific strain (e.g. C57BL/6J) when the metadata only supports the use of the parent strain (e.g. C57BL/6). We therefore added one sentence to the mouse strain prompt: ‘prefer the most general strain that is consistent with the evidence; only return a specific sub-strain if the text explicitly mentions the sub-strain by name or a vendor stock identifier '. This ‘specificity rule patch’ led to a significant but incomplete reduction in this class of errors (Fig. 3, ‘+spec rule’ columns; p = 0.006 for Sonnet, p = 0.0025 for GPT-4o).

We evaluated whether the ‘supporting evidence’ context output by the LLMs was accurate for four models. All four models score above 98% in the ‘normalized’ (lenient) evaluation (Supplementary Fig. S6). GPT-4o and Haiku were notably worse at producing verbatim quotes, tending to summarize.

**Figure 5:**
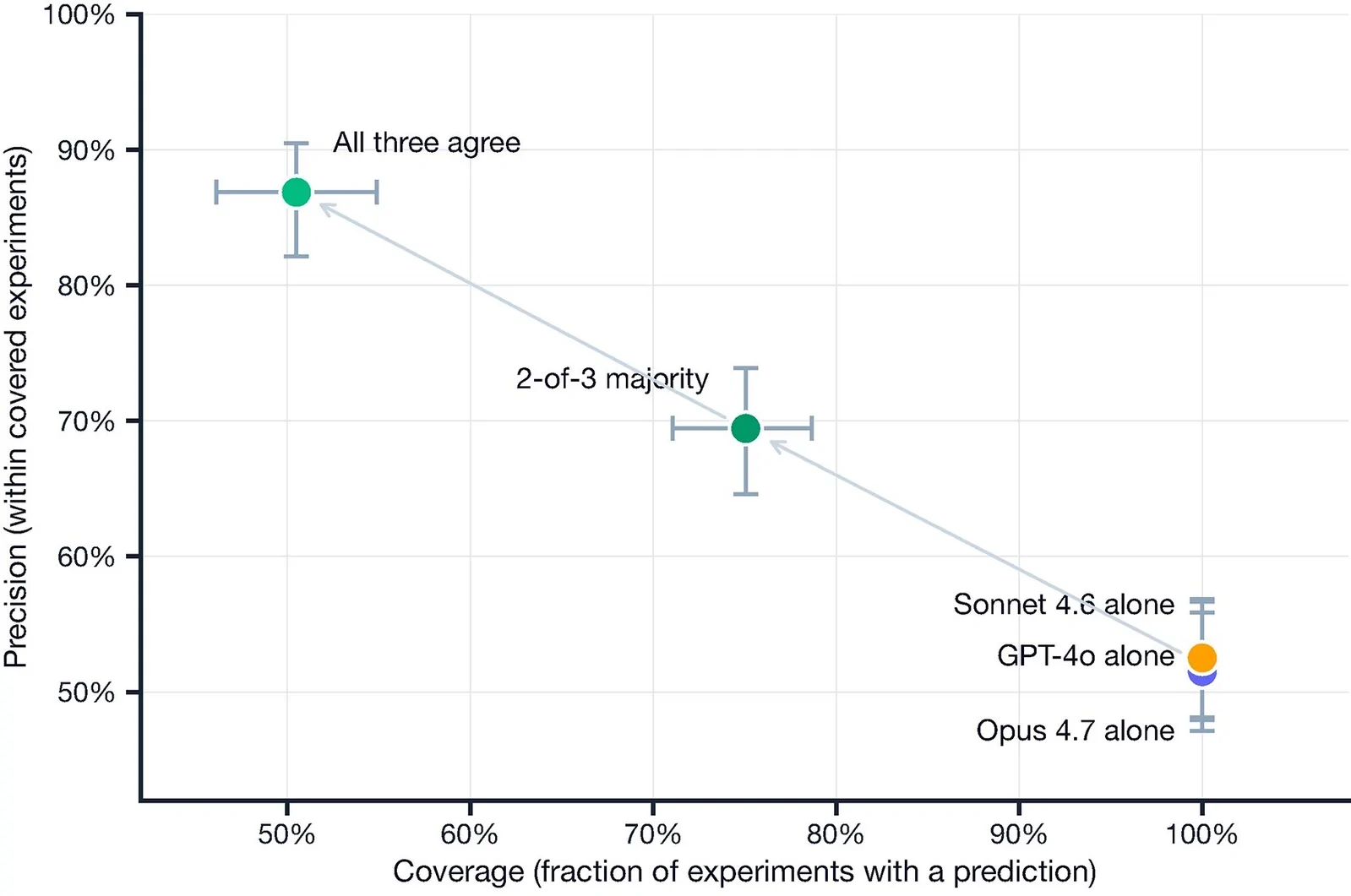
Cell line ensemble precision vs coverage. Independent frontier models converge less often on cell lines than on strains; intersecting their predictions trades coverage for precision. Error bars are 95% confidence intervals.

The specific hits and misses made by different high-performing LLMs were moderately to highly correlated on the strain task (Cohen’s kappa 0.68–0.85; 80–90% identical predictions considered as pairs; Supplementary Table S1). However, for the cell line task, the models were different enough (55–67% identical predictions; Supplementary Table S2) to raise the question of whether an ensemble would perform any better. While our work was in review, Mittal *et al*. [15] reported a system that uses internal arbitration between LLMs along with SapBERT, which may have some analogy to the ‘ensemble’ approach we piloted. We conducted a direct evaluation of their approach on the same 500-dataset sample, using GPT-4o as the underlying LLM (Methods). We found that MetaMuse-GPT-4o is conservative, making calls only 38% of the time but with relatively high precision (70.1%). In comparison, Our (ad hoc) three-model ensemble reaches even higher precision at the cost of some coverage (Fig. 5). Overall, our baseline approach still compares favorably given the tradeoffs between precision and recall, but these results suggest that proposer-arbitrator or other ensemble methods will be a productive way forward.

## Discussion

In this study, we systematically evaluated the performance of GPT-4o for entity-to-ontology annotation, focusing on two important sample descriptors commonly seen in transcriptomic experiments: mouse strains and cell lines. Using 9 390 manually curated experiments from Gemma and 5 230 associated publications across the two annotation tasks, we show that GPT-4o can accurately identify and normalize the majority of relevant entities. Non-LLM approaches turned out to perform less well. The choice of LLM does matter, but as it happens, GPT-4o remains a strong choice due to its low cost. Using the latest frontier models may bring small improvements at a much higher cost.

GPT-4o performed particularly well on the mouse strain annotation task, achieving correct annotation for 77% of experiments and high average recall and precision. Considering that the input text—consisting of the GEO experiment metadata and, when available, sections of the associated paper—was long, heterogeneous, and inconsistent, often containing discrepancies or typographic errors, we view this result as very promising.

Cell line annotation remains a challenging task, with only 59% of experiments annotated correctly. We believe this is partially due to the much larger Cell Line Ontology dictionary, consisting of over 46 000 terms (compared to 156 mouse strain ontology terms), as well as the high lexical complexity of cell line identifiers, frequently consisting of short, similar alphanumeric codes that pose a challenge even for human curators. In line with this, Riquelme-Garcia *et al*. [9] reported the lowest performance for CLO compared with the three other ontologies. In our hands, the RAG framework introduced errors arising from the retrieval and ranking of candidate ontology terms, and a hybrid dense + sparse method did not yield improvements. This remains a topic for further investigation. We also found that an ensemble of LLMs could improve precision, suggesting this approach should be explored as well.

In many cases GPT-4o’s (and other LLMs) errors closely resembled those made by human curators. They often arose from typographical mistakes in the GEO record, inconsistent naming or discrepancies between GEO metadata and the associated publications. Consistent with this observation, GPT-4o was more likely to make mistakes in experiments where string matching also failed to detect the correct mouse strains. We also observed occasional hallucinated annotations, where GPT-4o produced ontology terms not explicitly mentioned in the input text. Importantly, however, the supporting quotes from the input text returned by the model were reliably accurate, allowing errors to be readily identified during review.

Interestingly, GPT-4o identified over 200 errors in Gemma’s annotations (out of over 9 000), even though the original curation was performed manually by trained data curators using assistance tools for mapping free text entries to ontology terms and following strict guidelines. In addition, these annotations are often reviewed by a second curator and occasionally subjected to systematic quality audits. The errors often stemmed from inconsistent reporting within GEO metadata and/or the associated publications, problems that are very time-consuming and difficult for humans to catch. GPT-4o and the other high-performing LLMs were apparently able to overcome this by more effectively analyzing the entirety of the provided information.

There have been some other attempts to use LLMs for related tasks, some of which appeared since our preprint was posted. Most recently Mittal *et al*. [15] used an LLM to identify features such as cell types and tissues in GEO studies in a system called MetaMuse, which we included in our comparison. Hier, Do, and Obafemi-Ajayi [23] reported an F1 of 0.92 in a clinical features normalization task, using GPT-4o and RAG, well above BioBERT (closest in spirit to the SapBERT approach we evaluated and which MetaMuse also relies on). Huh [24] reported 57.6% exact matches on a challenging medical language mapping task, using GPT-4 (gpt-4–0613) and RAG. Dobbins [25] likewise reported superior performance using LLMs to augment other approaches. There are studies where LLMs have not fared as well. Riquelme-García *et al*. [9] found that without fine-tuning, the base models performed poorly at biological sample label normalization, with precision across models not reaching 20%. Because the LLMs were given only sample labels as input, the lack of broader context may have limited their ability to select the correct ontology identifier. In addition, since a RAG framework was not used in the study, the models often produced random identifiers that did not correspond to existing ontology terms. Kainer [7] investigated the ability of GPT-4o to annotate plant phenotype observations with ontology terms using several workflows, both with and without the RAG framework. Their RAG-based workflows ‘descriptor to embedding’ and ‘descriptor to concept to embedding’, which most closely resemble our mouse strain and cell line annotation approaches, respectively—achieved a highest recall of 0.517 and a highest precision of 0.397. A possible explanation for the lower performance in their study is the greater complexity of the annotation task, which required identifying and mapping multi-word phenotype concepts rather than single-entity mentions. On the other hand, they gave the model only single-sentence inputs, limiting the volume of text to be analyzed.

We were a little surprised at how poorly some of the other approaches performed, for example with regular expressions outperforming text2term. However, we must stress that our task is not simply normalizing strings to ontology terms, it is to identify which strain or cell line was used in the expression analysis, which is often not obvious in the GEO record or even the publication. An author statement such as ‘We used a strain derived from C57BL6’ should not lead to an annotation of C57BL6. A simple normalization approach would generate a false positive. We believe that LLMs can help fill this gap. Still, we can comment on the normalization tool failures. We believe text2term puts too much weight on the length of a term. Curators rarely use ‘C57BL’ but text2term favors it over ‘C57BL6’, and, in fact, it proposed C57BL in 297/500 cases examined, all but one of which were false positives. BM25 was much better performing, but apparently suffered from over-tokenizing, making the annotation ‘A/J’ in 165/500 cases (correctly only three times), apparently because it tokenizes to the letters A and J. SapBERT also performed weakly, and we can relate our results to Mittal *et al*. [15], who used SapBERT for ontology term normalization. Mittal *et al*. also reported problems with normalizing cell lines (55% accuracy), but they did not report on LLM-guided approaches for that step. In other settings, these methods can do better and we suspect some tuning would recover some performance.

Our study has several limitations. Although Gemma’s transcriptomic experiments are carefully curated, the annotations do not constitute a definitive ground truth, and in fact, even GEO records and publications should be treated with caution. This is illustrated by the fact that Gemma contains curation errors, many of which were propagated from the sources. Although these were corrected before calculating final accuracy measures for GPT-4o, some disagreements between GPT-4o and the reference annotations may still reflect limitations of the reference data rather than model errors. Second, for the cell line annotation task, performance depended on the embedding-based retrieval of candidate ontology terms. Failures to retrieve the correct candidate prevented correct ontology normalization even when the entity mention was correctly identified in the first stage, meaning the measured accuracy does not reflect the performance of the language model alone. Finally, we did not measure inter-annotator agreement among human curators, and therefore cannot directly compare model performance to the natural variability present in manual curation.

Overall, our findings indicate that GPT-4o and other currently available LLM models cannot fully replace a human curator for the entity-to-ontology annotation task. However, they show clear promise as assistive tools that can improve both the efficiency and quality of annotation and we also identified ways to improve the methods. An effective curation workflow would adopt a human-in-the-loop model, in which LLMs perform initial screening of input text, identify and normalize entities or concepts, and provide results together with the supporting quotes for validation by human curators. Such an approach could help scale curation efforts for rapidly growing biomedical databases and repositories while preserving human oversight. Future work could explore tighter integration of LLMs into interactive curation workflows, the potential benefit of fine-tuning, improved retrieval strategies for large ontologies, and extension of this approach to additional metadata categories and experimental modalities.

## Supplementary Material

baag041_Supplemental_Files

## Data Availability

The spreadsheets listing Gemma’s experiments used for evaluation of mouse strain and cell line annotation tasks, along with manual and predicted annotations, GPT-4o-generated supporting quotes, ontology URIs, performance metrics, curators’ notes and other details are available at https://github.com/PavlidisLab/GPT_annotate. The repository also contains GPT-4o prompts and scripts for running the experiments and evaluating the models’ performance.
