# Supplementary material for: Application of large language models to the annotation of cell lines and mouse strains in genomics data
Source: Database (Oxford). 2026 Jul 22;2026:baag041. doi: 10.1093/database/baag041 (PMC13389303; doi:10.1093/database/baag041)
Supplement: baag041_Supplemental_Files [file baag041_supplemental_files.zip › Supplementary_figures.pdf]

Supplementary Figures

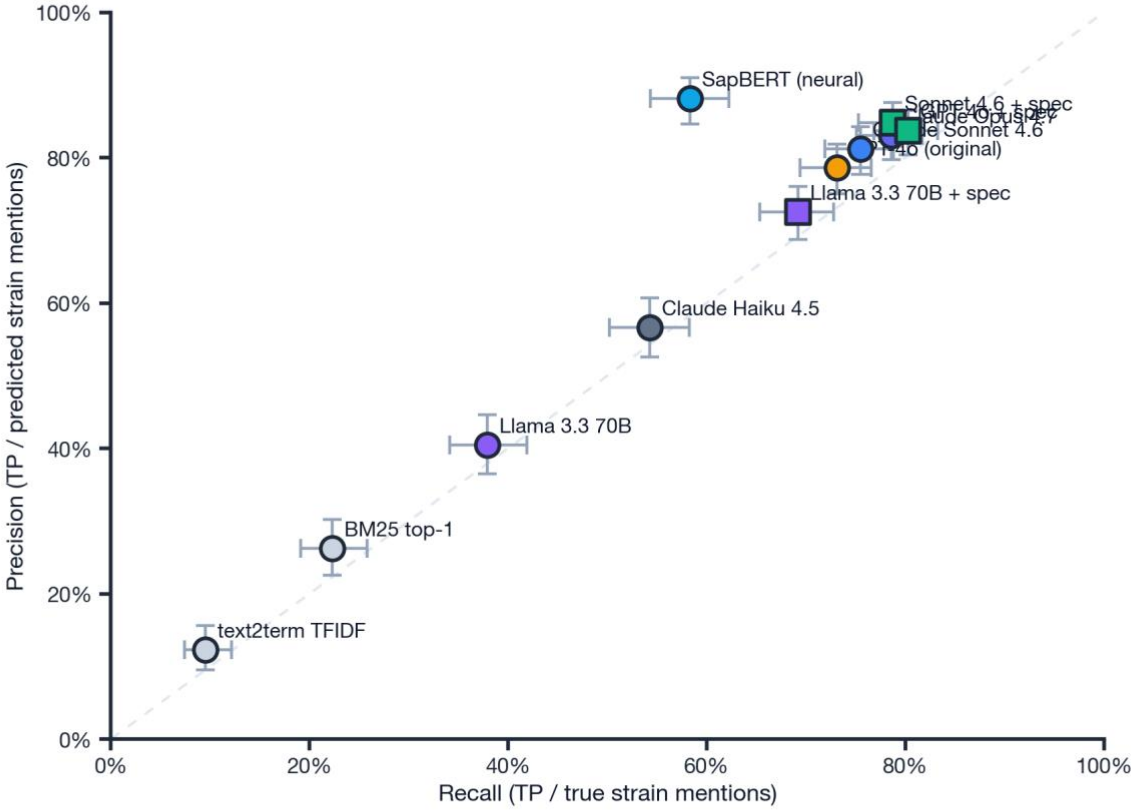

Supplementary Figure S1: Strain task strain-level micro precision vs recall. Each point aggregates true positives, false positives and false negatives across the 500-GSE sample at the individual-strain level; multi-strain GSEs contribute each strain separately. Every method sits above the y = x diagonal: they miss strains in multi-strain GSEs more often than they fabricate strains. Error bars are 95% confidence intervals over the strain-mention totals.

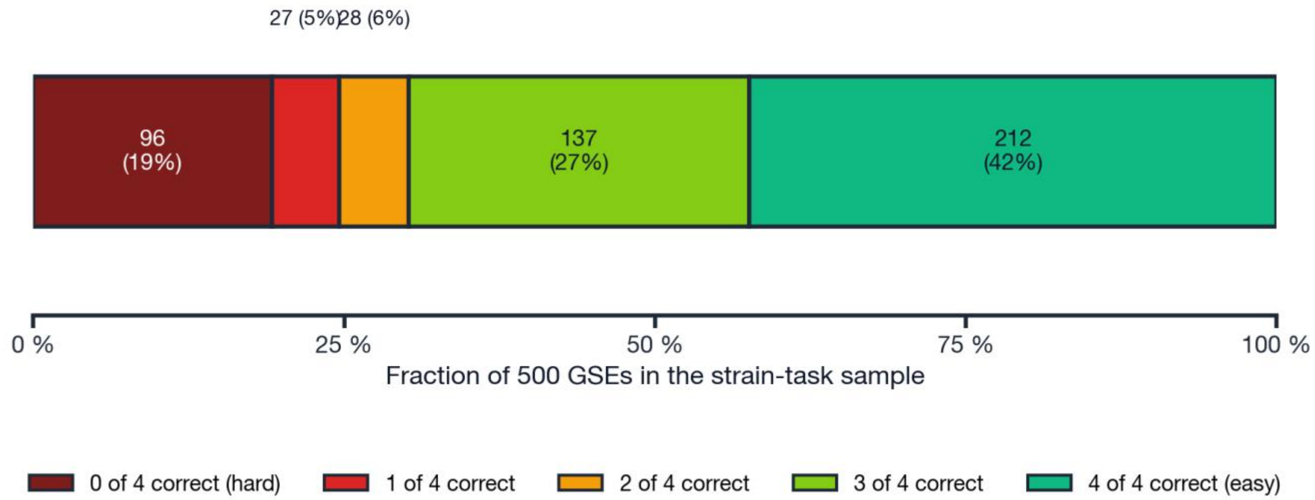

Supplementary Figure S2: Strain task correctness overlap across the four frontier LLMs (Sonnet 4.6, Opus 4.7, GPT-4o, Haiku 4.5). Evaluated on the same 500-GSE sample. Each GSE is classified by how many of the four models annotated it correctly. The 19% all-wrong block isolates intrinsically ambiguous cases; the 42% all-correct block is the easy floor. Most of the model-to-model spread is decided on the middle 38% where models disagree.

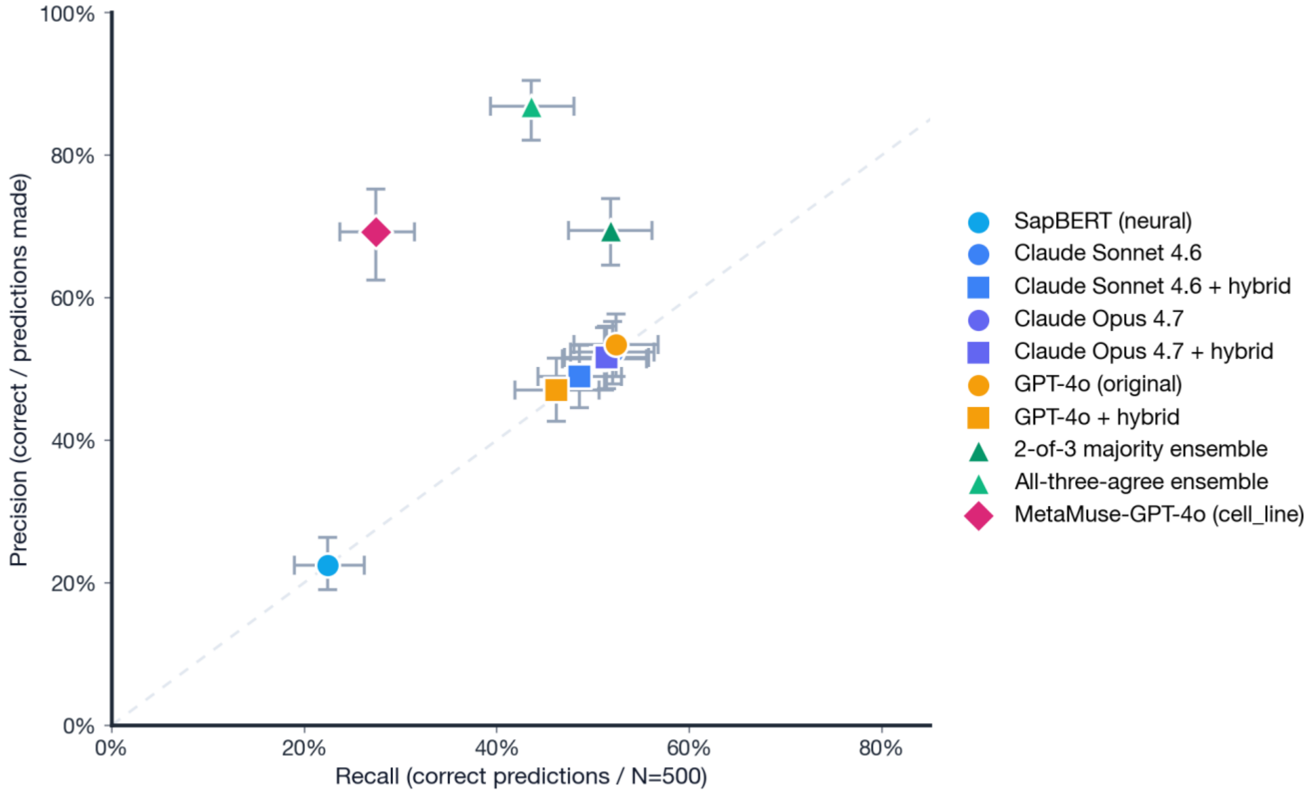

Supplementary Figure S3: Cell-line task precision vs recall (cross-walk-aware). Stage-1+2 LLMs and SapBERT cover ~98–100% of GSEs, so they sit on the diagonal. Abstention-based systems lift precision above the diagonal at the cost of recall: ensembles (triangles; 2-of-3 majority and all-three-agree intersection) and MetaMuse (diamond). Squares: + BM25 hybrid retrieval. Dashed line: P = R.

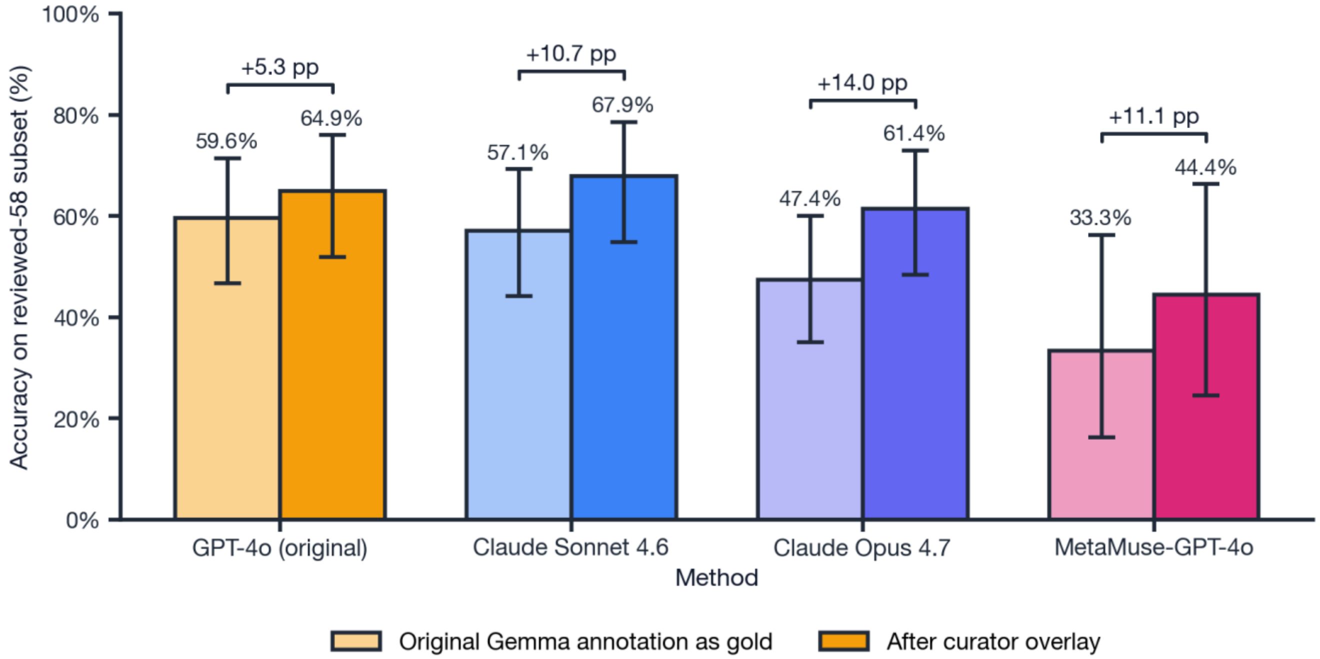

Supplementary Figure S4: Cell-line task accuracy after curator review. A single curator reviewed 156 new predictions across 58 GSEs (71 judged correct, 78 wrong, 7 uncertain). Bars compare the original Gemma-as-gold accuracy to the post-overlay accuracy on the reviewed 58-GSE subset. Error bars are 95% confidence intervals.

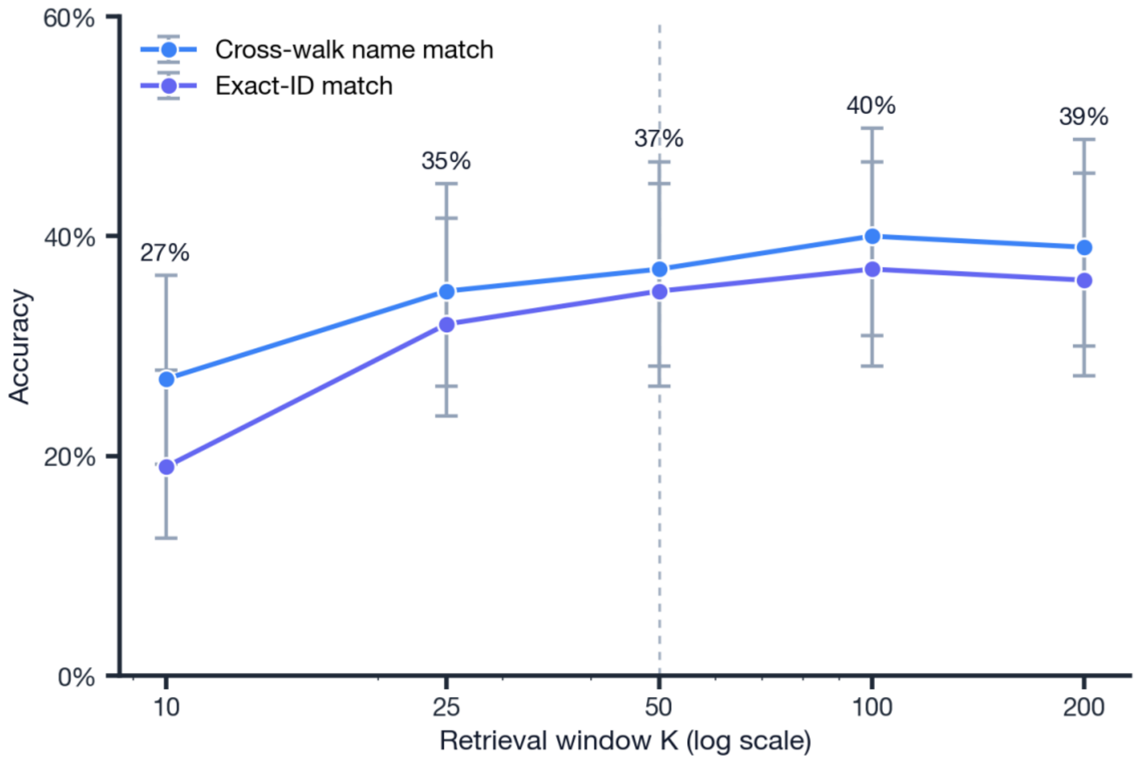

Supplementary Figure S5: Cell-line accuracy vs retrieval window K. Sonnet 4.6, 100-experiment subset. Vertical dashed line marks K = 50, the value used in the main results. Error bars are 95% confidence intervals.

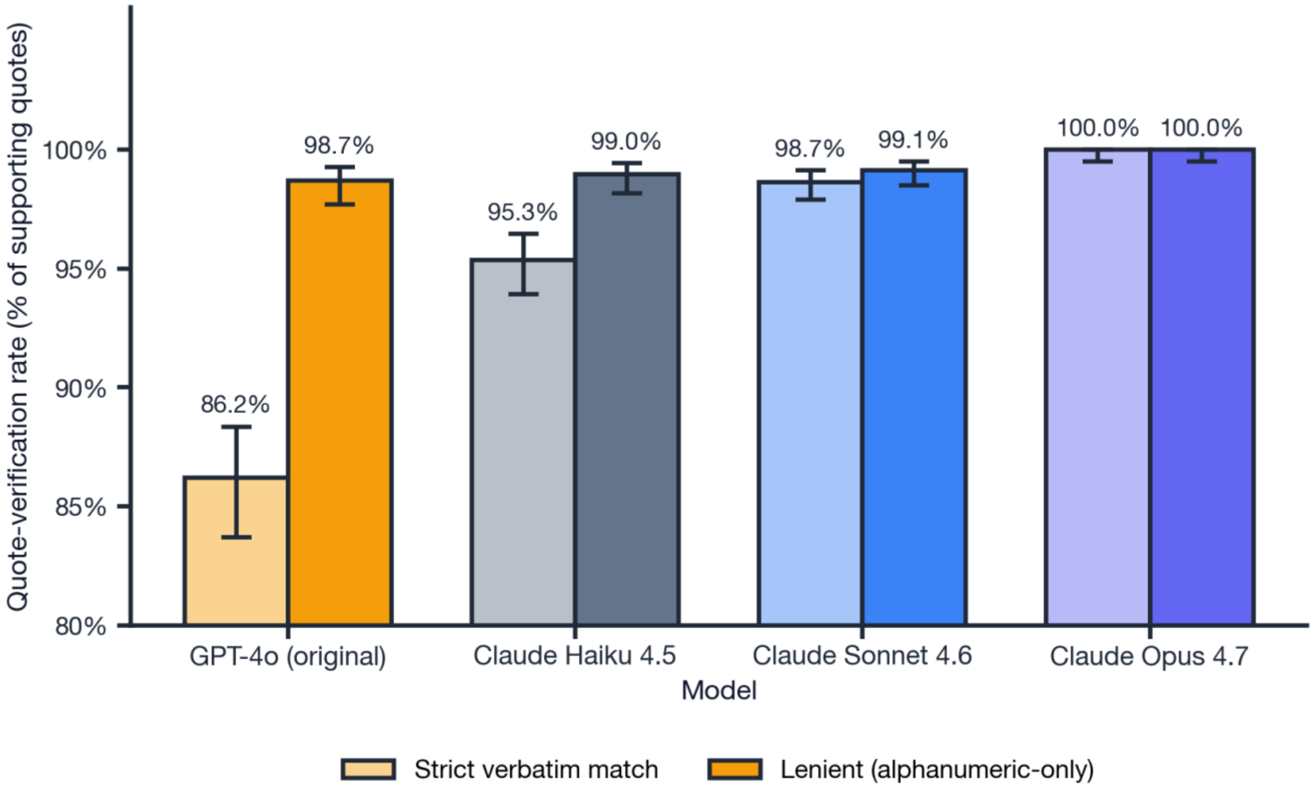

Supplementary Figure S6: Quote-grounding rates on the strain task. Verbatim substring match (strict) vs alphanumeric-only normalization (lenient). All four models verify ≥ 98.7% of their quotes under the lenient metric — the strict-match gap is cosmetic, not fabrication. Error bars are 95% confidence intervals.
